# Analgesic effect of erector spinae plane block in adults undergoing laparoscopic cholecystectomy: a systematic review and meta-analysis of randomized controlled trials

**DOI:** 10.1186/s12871-023-01969-6

**Published:** 2023-01-06

**Authors:** Xiaoli Yang, Yu Zhang, Yong Chen, Mingxing Xu, Xue Lei, Qiang Fu

**Affiliations:** Department of Anesthesiology, The Third People’s Hospital of Chengdu, Chengdu, Sichuan China

**Keywords:** Erector spinae plane block, Postoperative pain, Adult, Meta-analysis

## Abstract

**Background:**

Laparoscopic cholecystectomy is the gold standard surgical procedure for treating gallstone disease. Despite it being minimally invasive, various medications and methods are used to alleviate postoperative pain, and some patients still experience moderate-to-severe pain. This is a crucial problem that must be solved to avoid chronic pain. As part of postoperative multimodal analgesia, regional block is being increasingly applied in surgery under ultrasound guidance. We aimed to evaluate the analgesic effect of erector spinae plane block in adult patients undergoing laparoscopic cholecystectomy.

**Methods:**

PubMed, Cochrane Library, EMBASE, and Web of Science were searched for randomized controlled trials investigating the efficacy of erector spinae plane block on postoperative pain after laparoscopic cholecystectomy. The primary outcome was the postoperative pain score. The secondary outcomes were the cumulative intraoperative and postoperative opioid consumption at 24 h, incidence of postoperative nausea and vomiting, and shoulder pain after surgery. The results were pooled using the fixed- or random-effects model with Review Manager 5.3.

**Results:**

Fifteen randomized controlled trials involving 947 patients were included in the analysis. Postoperative pain score in the erector spinae plane block group was lower than that in the control group at postoperative 12 h (MD − 0.81, 95% CI − 1.1 to − 0.51, *p* < 0.00001) and 24 h (MD − 0.41, 95% CI − 0.62 to − 0.19, *p* = 0.0002). Cumulative opioid consumption was lower in the erector spinae plane block group than in the control group at postoperative 24 h (MD − 7.88, 95% CI − 10.17 to − 5.58, *p* < 0.00001). The erector spinae plane block group also experienced a lower incidence of postoperative nausea and vomiting than the control group. Opioid consumption and the incidence of postoperative nausea and vomiting were similar between the erector spinae plane block group and other block groups, including the oblique subcostal transversus abdominis plane block and quadratus lumborum block groups.

**Conclusions:**

Ultrasound-guided erector spinae plane block provides effective postoperative analgesia in adults undergoing laparoscopic cholecystectomy.

**Supplementary Information:**

The online version contains supplementary material available at 10.1186/s12871-023-01969-6.

## Background

Laparoscopic cholecystectomy (LC) is the most commonly performed surgical procedure for cholelithiasis management. Although it is minimally invasive and is associated with a shorter hospital stay and faster recovery than open surgery, some patients still experience moderate-to-severe postoperative pain [1–3]. Acute pain after LC consists of somatic, parietal, and referred pain caused by trocar insertion, gall bladder resection, carbon dioxide insufflation, and other factors [4, 5]. If handled improperly, some patients (3–56% according to different studies) may experience prolonged or chronic pain [6]. Therefore, various drugs have been used worldwide to relieve postoperative pain.

Lately, under ultrasound guidance, regional blocks have been performed more accurately, providing better postoperative analgesia management [7, 8]. The erector spinae plane block (ESPB), first described by Forero et al. in 2016 for the treatment of thoracic neuropathic pain [9], has proven effective for acute pain control in abdominal, spinal, breast, and other surgeries [10]. As it can block sympathetic nerve fibers and the ventral rami of spinal nerves [11–13]and is easier to perform and safer than paravertebral block, it is quickly gaining popularity among anesthesiologists and applied in various surgeries as a part of multimodal analgesic regimens.

In the past 6 years, an increasing number of studies have been published to support the efficacy of ESPB, including case reports, clinical trials, and meta-analyses involving different surgical types [14, 15]. Hence, we aimed to evaluate the analgesic effect of ESPB in adults undergoing LC and compare it with other regional blocks.

## Methods

The Preferred Reporting Items for Systematic Reviews and Meta-Analyses (PRISMA) was followed to perform this meta-analysis [16]. This meta-analysis was conducted using a predesigned protocol registered with PROSPERO (registration number: CRD42022336837).

### Systematic search and inclusion criteria

All randomized controlled trials (RCTs) comparing ESPB with no block or other regional blocks in adults undergoing LC were included. Electronic databases including PubMed, Cochrane Library, EMBASE, and Web of Science were comprehensively searched for RCTs published before May 30, 2022. Literature search was conducted using a combination of medical subject headings and entry terms, including “(Laparoscopic or Celioscopic) and Cholecystectom*”, “erector spinae plane block”, “erector spinae plane”, “ESPB”, “ESP”, and “ESB”. A detailed search strategy for each database is available in Additional File 1. In addition, the reference lists of all included studies were checked for any potential additional publications.

### Selection of included studies and data extraction

Two experienced authors (Xiaoli Yang and Yu Zhang) independently screened the titles and abstracts of each article to eliminate repeated and irrelevant studies. The full texts of potentially eligible studies were then reviewed, and articles that meet the eligibility criteria were included. The data were extracted by two independent authors (Xiaoli Yang and Yu Zhang). Disagreements were resolved through discussion. If necessary, a third reviewer participated in the discussion to reach a consensus. The following data were extracted: first author, year of publication, sample size, type of surgery, ESPB target spine level and local anesthetics, control group technique, intraoperative opioid consumption, postoperative analgesia protocol, postoperative pain score and opioid consumption, postoperative nausea and vomiting(PONV), shoulder pain, and block-related complications. Data presented in the form of graphs were extracted using Plot Digitizer, a graph digitizing software.

### Risk-of-bias assessment

Two reviewers (Xiaoli Yang and Yu Zhang) independently assessed the quality of included trials using the Cochrane Collaboration tool [17]. Each included trial was assessed as low risk, unclear, or high risk in the following seven domains: random sequence generation, allocation concealment, blinding of participants and personnel, blinding of outcome assessment, incomplete outcome data, selecting reporting, and other sources of bias.

### Primary outcome

Postoperative pain score at postoperative 12 and 24 h.

### Secondary outcomes

Cumulative intraoperative and postoperative opioid consumption at 24 h, incidence of PONV, shoulder pain, and other block-related complications after surgery.

### Meta-analysis

The meta-analysis was conducted using Review Manager (version 5.3; Nordic Cochrane Centre). For dichotomous variables, the risk ratio (RR) and 95% confidence interval (CI) were calculated. For continuous variables, the mean difference (MD) and 95% CI were calculated. If continuous variables were expressed as median and range (minimum to maximum or interquartile range), Luo and Wan’s formula was used to estimate the mean and standard deviation [18, 19]. If the standard deviation was missing, we used that of RCTs that conducted the same intervention for calculation. Higgins’s *I*^*2*^ statistical test was used to assess the statistical heterogeneity of the pooled results [20]. *I*^*2*^ between 0 and 25% was interpreted as no heterogeneity, 25–50% as low heterogeneity, 50–75% as moderate heterogeneity, and 75–100% as high heterogeneity. The fixed- or random-effects model was selected according to the level of heterogeneity. If *I*^*2*^ was < 50%, the fixed-effects model was selected, whereas if *I*^*2*^ was > 50%, the random-effects model was selected. A trial sequential analysis of the primary outcome was performed to confirm whether the sample size was sufficient and the results were stable or not [21]. Statistical significance was set at *P* < 0.05.

## Results

**Fig. 1:**
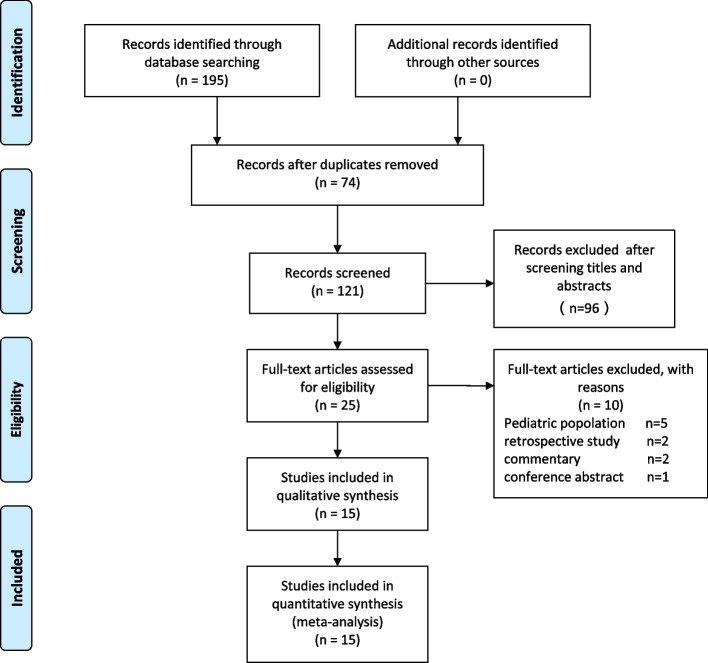
Flow diagram of the included and excluded studies

**Fig. 2:**
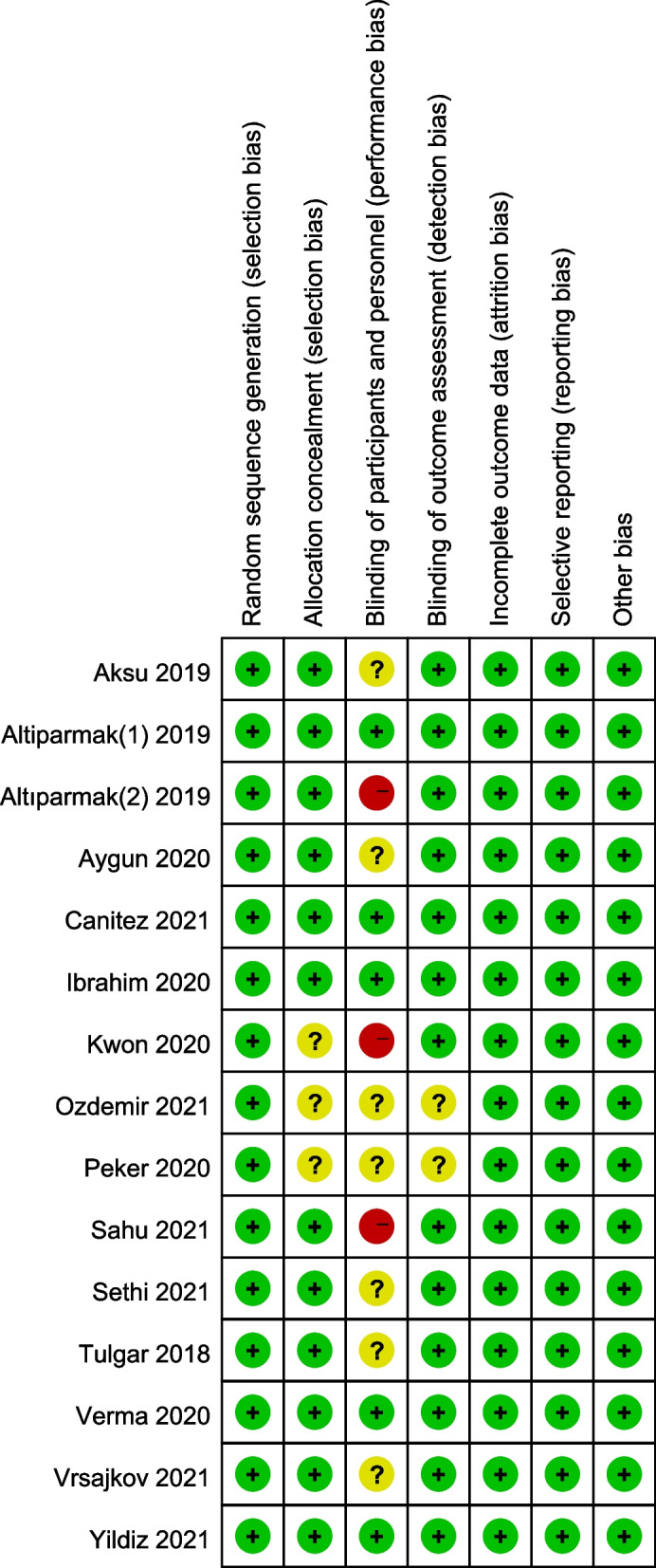
Risk-of-bias summary: each risk-of-bias item for each included study

A literature search identified 195 records based on the eligibility criteria, and 74 articles were excluded for duplication. After screening the titles and abstracts, 96 studies were excluded, and the full text of the remaining 25 potentially eligible studies was reviewed. Among them, five studies were excluded because of the pediatric population, two articles were excluded due to retrospective study, two for commentary, and one for conference abstract. Therefore, 15 RCTs involving 947 patients were included in this systematic review and meta-analysis [22–36], and the detailed flow diagram is presented in Fig. 1. The characteristics of the included studies are summarized in Table 1. The risk of bias is shown in Fig. 2.

**Table 1:** Characteristics of included studies

Study	ESPB(*N*)	Control(*N*)	ESPB local anesthetic	Block level	Postoperative analgesia
Asku 2019 [22]	23	23	0.25% BUPI 20 ml	T8	Paracetamol 1 g iv; tramadol 100 mg; morphine PCA
Altiparmak(1)2019 [23]	21	20	0.25% BUPI 20 ml × 2	T7	tramadol PCA; morphine 4 mg iv as needed
Canıtez 2021 [24]	41	41	0.5% BUPI 7.5 ml + 2% LDC 2.5 ml + NS 10 ml × 2	T8	Paracetamol 1 g iv q8h; tenoxicam 20 mg iv q12h; tramadol 1 mg/kg as needed
Kwon 2020 [25]	26	27	0.20% ROPI 20 ml × 2	T7	Fentanyl 0.4μg/kg as needed in PACU; dexketoprofen 50 mg, tramadol 50 mg or meperidine 25 mg as needed
Sethi 2021 [26]	33	33	0.25% Levo-BUPI 20 ml × 2	T7	Paracetamol 1 g iv q8h;tramadol PCA; fentanyl 30μg iv as needed
Tulgar 2018 [27]	15	15	0.375% BUPI 20 ml × 2	T9	Fentanyl 25μg as needed in PACU; tramadol PCA; paracetamol 1 g iv q8h; diclofenac Na 75 mg im and meperidine 50 mg iv as needed
Verma 2020 [28]	42	42	0.375% BUPI 20 ml × 2	T7	Paracetamol 1 g iv q8h; aqueous diclofenac 75 mg iv as needed
Vrsajkov 2021 [29]	30	30	0.25% Levo-BUPI 20 ml + DEX 2 mg × 2	T7	Acetaminophen 1 g iv q8h; ketorolac 30 mg q8h; tramadol 1 mg/kg as needed
Yildiz 2021 [30]	34	34	0.5% BUPI 10 ml + 2% LDC 5 ml + NS 5 ml × 2	T8	Acetaminophen 1 g iv; tenoxicam 20 mg iv; tramadol 50 mg as needed
Peker 2020 [31]	38	44	0.25% BUPI 20 ml × 2	T7	Paracetamol 1 g iv; tenoxicam 20 mg iv; tramadol 1 mg/kg as needed
Altiparmak(2)2019 [32]	34	OSTAPB(34)	0.375% BUPI 20 ml × 2	T7	Dexketoprofen trometamol 50 mg iv;tramadol PCA; morphine 4 mg iv as needed
Ibrahim 2020 [33]	21	Control(21) OSTAPB(21)	0.25% BUPI 20 ml × 2	T8	Fentanyl 15-20μg or morphine 1-2 mg or pethidine 15-30 mg iv in PACU;paracetamol 1 g iv q6h; morphine PCA
Ozdemir 2021 [34]	32	OSTAPB(32)	0.25% BUPI 10 ml + 2% PRI 10 ml × 2	T7	Paracetamol 1 g iv; tenoxicam 20 mg iv; paracetamol 15 mg/kg iv q6h; fentanly PCA; meperidine 25 mg as needed;
Sahu 2021 [35]	30	OSTAPB(30)	0.20% ROPI 20 ml + DEX 4 mg × 2	T7	Paracetamol 1 g iv q6h; tramadol 1 mg/kg and diclofenac 75 mg iv infusion as needed
Aygun 2020 [36]	40	QLB(40)	0.5% BUPI 15 ml + 2% LDC 5 ml + NS 10 ml × 2	T9	Paracetamol 1 g iv; tenoxicam 20 mg iv; morphine PCA

*PCA* patient-controlled analgesia, *ESPB* erector spinae plane block, *(O)STAPB* (oblique) subcostal transversus abdominis plane block, *QLB* quadratus Lumborum Block, *BUPI* bupivacaine, *ROPI* ropivacaine, *LDC* lidocaine, *NS* normal saline, *DEX* dexamethasone, *PRI* prilocain

### ESPB vs. control

**Fig. 3:**
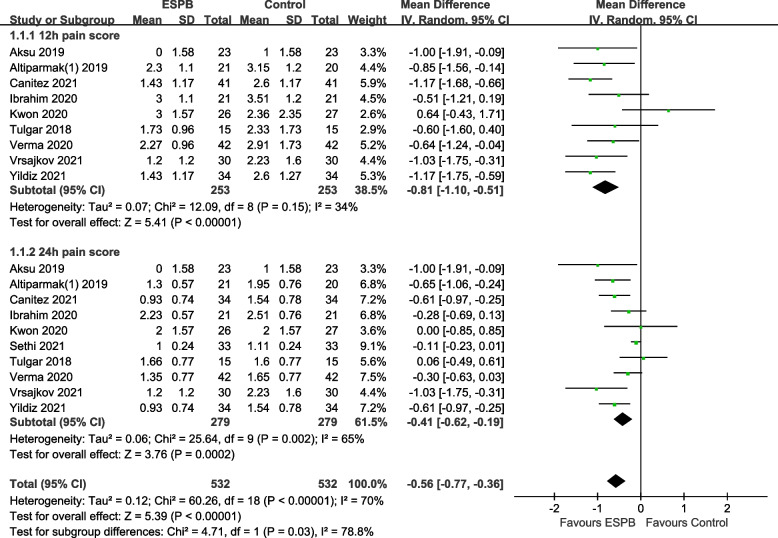
Forest plot for postoperative pain scores. Pain score at postoperative12 and 24 h was significantly lower in the ESPB group than in the control group

**Fig. 4:**
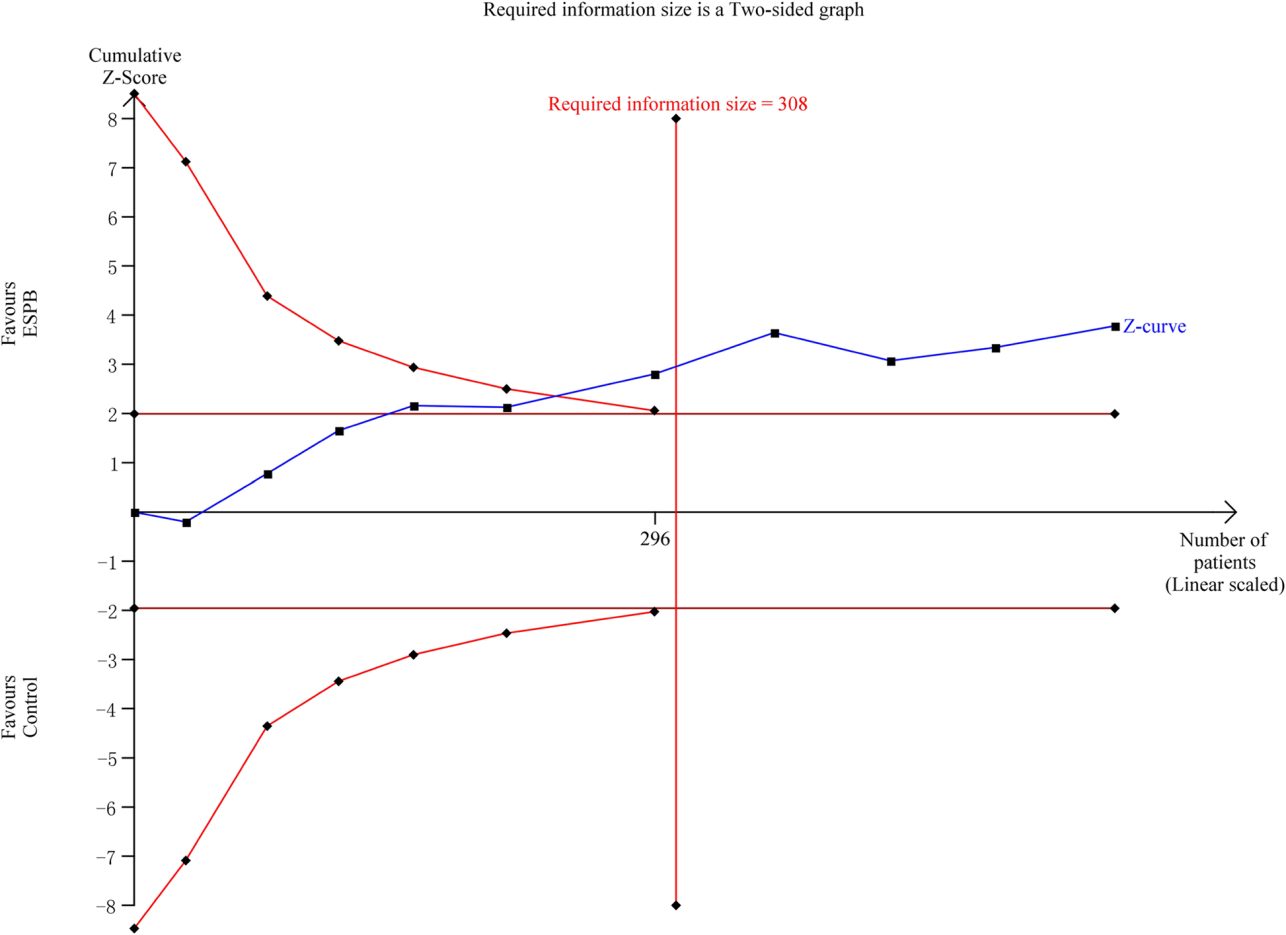
Trial sequential analysis for pain score at postoperative 24 h. The cumulative Z-curve crosses the monitoring boundary curve, indicating firm evidence that the ESPB group showed superior findings than the control group

Ten RCTs including 612 patients reported postoperative pain scores using a numerical rating scale or visual analog scale at a predefined time point. The pooled results demonstrated that ESPB significantly lowered the pain score compared with the control group at postoperative 12 h (MD − 0.81, 95% CI − 1.1 to − 0.51, *p* < 0.00001) and 24 h (MD − 0.41, 95% CI − 0.62 to − 0.19, *p* = 0.0002). Low-to-moderate level of heterogeneity was observed (Fig. 3). The trial sequential analysis was performed on the pain score at postoperative 24 h, indicating that firm evidence was reached regarding the contribution of ESPB to decrease the pain score at postoperative 24 h. The cumulative Z-curve crosses the monitoring boundary curve before the accumulated information reaches the required information size, indicating that the relief of postoperative pain by ESPB has been proven (Fig. 4).

**Fig. 5:**
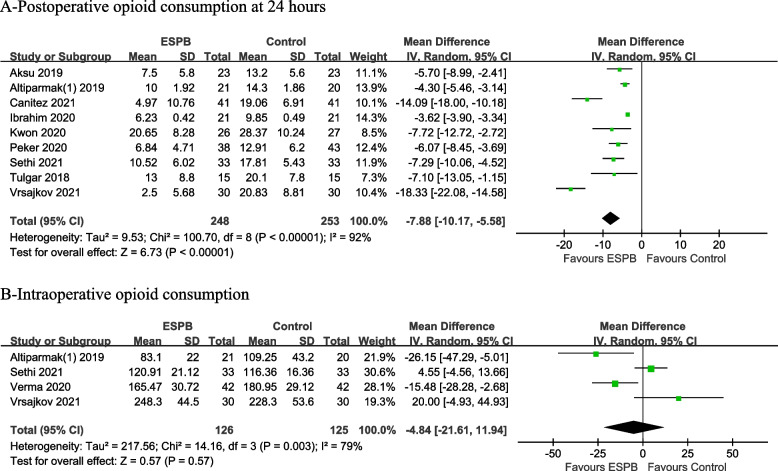
Forest plot for postoperative and intraoperative opioid consumption. Opioid consumption at postoperative 24 h was significantly lower in the ESPB group than in the control group. Intraoperative fentanyl consumption was comparable between the ESPB and control groups

Postoperative opioid consumption was reported in nine RCTs involving 501 patients. Among them, tramadol was used in six RCTs, morphine in two RCTs, and fentanyl in one RCT. To facilitate data analysis, tramadol and fentanyl were converted to morphine-equivalent doses based on previous studies suggesting that intravenous administration of 100 mg tramadol or 100 μg fentanyl was equivalent to 10 mg morphine. The results showed that opioid consumption at postoperative 24 h was significantly lower in the ESPB group than in the control group (MD − 7.88, 95% CI − 10.17 to − 5.58, *p* < 0.00001). High heterogeneity was observed (Fig. 5a).

Four RCTs assessed the intraoperative opioid consumption. Unexpectedly, no significant difference was noted between the ESPB and control groups (MD − 4.84, 95% CI − 21.61 to 11.94, *p* = 0.57). High heterogeneity was observed (Fig. 5b).

**Fig. 6:**
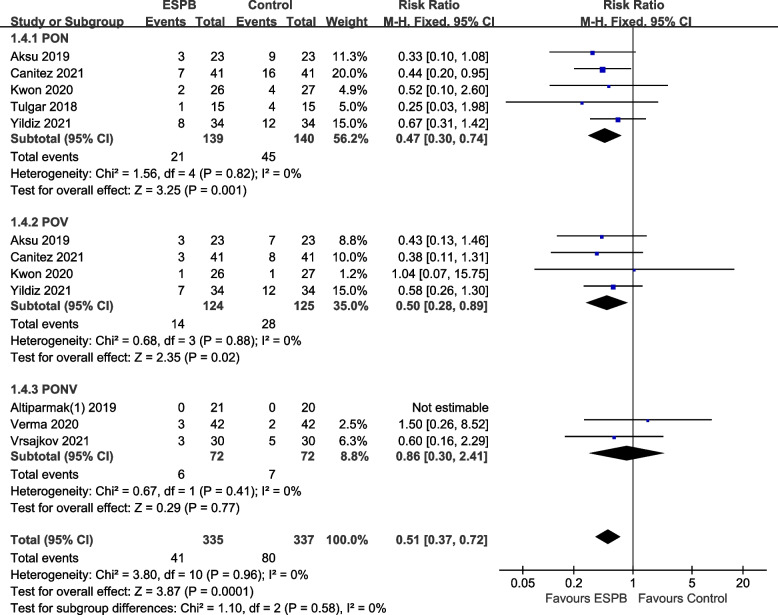
Forest plot for postoperative nausea and vomiting (PONV). Incidence of PONV was significantly lower in the ESPB group than in the control group

Five RCTs reported postoperative nausea (PON), and four RCTs reported postoperative vomiting (POV). The results showed that ESPB reduced the incidence of PON (RR 0.47, 95% CI 0.3 to 0.74, *p* = 0.001) and POV (RR 0.5, 95% CI 0.28 to 0.89, *p* = 0.02). No heterogeneity was observed among the studies. In one study included in this meta-analysis, no patient experienced PONV. Two studies reported that PONV showed no significant differences between the two groups (RR 0.86, 95% CI 0.3 to 2.41, *p* = 0.77; Fig. 6).

**Fig. 7:**
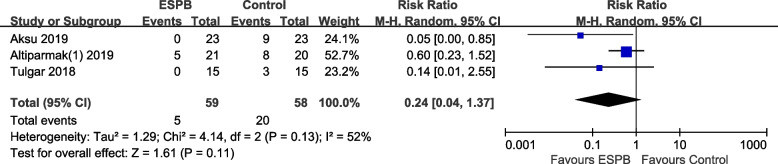
Forest plot for postoperative shoulder pain. Shoulder pain was comparable between the ESPB and control groups

Three RCTs assessed LC-related shoulder pain, and the results showed no significant difference between the ESPB and control groups (RR 0.24, 95% CI 0.04 to 1.37, *p* = 0.11; Fig. 7).

### ESPB vs. other blocks

**Fig. 8:**
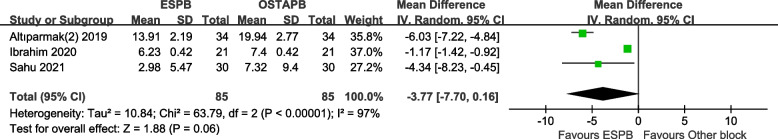
Forest plot for postoperative opioid consumption. Opioid consumption at postoperative 24 h was comparable between the ESPB and OSTAPB groups

**Fig. 9:**
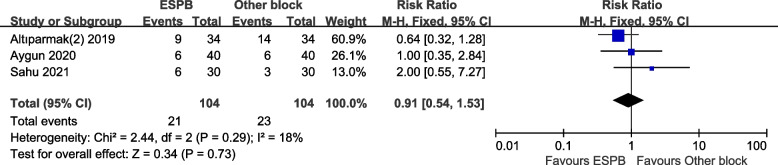
Forest plot for postoperative nausea and vomiting (PONV). Incidence of PONV was comparable between the ESPB and other groups

Three RCTs reported the postoperative opioid consumption between the ESPB and oblique subcostal transversus abdominis plane block (OSTAPB) groups, and one study assessed this outcome between the ESPB and quadratus lumborum block (QLB). Pooled results suggested that no significant difference in the requirement of opioids between the ESPB and OSTAPB groups (MD − 3.77, 95% CI − 7.7 to 0.16, *p* = 0.06; Fig. 8). PONV was reported in three RCTs, and no significant difference was observed (RR 0.91, 95% CI 0.54 to 1.53, *p* = 0.73; Fig. 9).

### Block-related complications

All included studies were carefully reviewed, except for one study that reported intraoperative bradycardia in two patients in the ESPB group and in one patient in the OSTAPB group, with no significant difference. Other intraoperative and postoperative adverse reactions or complications, including bleeding, hematoma, subcutaneous emphysema, pneumothorax, local anesthetic toxicity, and pruritus, were not reported. This may have resulted from the widespread use of ultrasound. Ultrasound facilitates the accurate identification of the target fascial planes and precise delivery of local anesthetics for safer conduct of the block.

## Discussion

Our meta-analysis demonstrated the clinical role of ESPB in postoperative pain control in adults undergoing LC. Postoperative pain scores at 12 and 24 h and opioid consumption during the first 24 h were lower in the ESPB group than in the control group. Evidence indicates that ESPB also reduced the incidence of PONV compared with the control group. However, although ESPB was implemented, intraoperative opioid consumption was not reduced during surgery compared with that in the control group. In addition, more evidence is needed in the future to compare the analgesic effects of ESPB and OSTAPB in LC.

The prevention and management of postoperative acute pain have been a worldwide issue worthy of attention that need to be addressed. Over the last few decades, opioids have been widely used in the management of surgery-related acute pain. Although opioids relieve pain in humans, they also cause problems. Addiction, chronic pain, prolonged length of hospital stay, related morbidity and mortality, and many adverse reactions to opioids strongly force us to face and look for new ways to manage pain [37]. Various regional blocks have recently been applied in surgery to achieve desired pain control and reduce opioid consumption. ESPB is a relatively novel regional block, and although the mechanism is still controversial, many clinical trials have confirmed its effectiveness in pain control and is being quickly applied as a part of multimodal postoperative analgesia. A previous meta-analysis by Koo et al. revealed that the ESPB group had lower the pain scores at postoperative 12 h than the control group, but no significant difference was noted at 24 h [38]. In our updated analysis, ESPB reduced the pain score at postoperative 24 h. To our knowledge, this is the first meta-analysis to show the long-lasting analgesic effect of ESPB at 24 h in LC. Postoperative opioid consumption was also lower than that in the control group, which is consistent with the findings of previous studies [39–41]. Unexpectedly, no significant difference was noted in the intraoperative opioid consumption between the ESPB and control groups. Among them, fentanyl was administered during the induction and maintenance of anesthesia; the surgery time in these studies ranged from 60 to 100 min, and addtional fentanyl may be administered in both groups. Further studies adopting opioid infusion, rather than single injection, may detect differences in intraoperative opioid consumption.

Postoperative nausea and vomiting are two common adverse events, with an estimated incidence of 30%; in high-risk patients, it can be as high as 80% [42]. The management of PONV is complex. In the fourth consensus guideline for the management of PONV, opioids were recognized as a risk factor for PONV in adults and showed dose dependency [43]. High-level evidence recommends reducing opioid use and combining multimodal analgesia, such as regional blocks, to prevent PONV [44, 45]. A previous meta-analysis by Daghmouri et al. reported no significant difference between the ESPB and control groups, but only included five RCTs [46]. Our meta-analysis found that ESPB can reduce the incidence of PON and POV after surgery, which is consistent with the results of Koo’s study [38]. Regional blocks such as ESPB, as mentioned above, possibly reduce the incidence of nausea and vomiting by reducing opioid consumption.

Compared with other blocks such as OSTAPB and QLB, no significant difference was observed between ESPB and OSTAPB in terms of postoperative opioid consumption, which is consistent with the findings of Koo’s study [38]. Although the exact mechanism of ESPB is unclear, the available evidence shows that the physical spread of local anesthetic may be the most likely mechanism. Although the extent of the spread of local anesthetic remains controversial, most studies have shown that local anesthetic may spread to the paravertebral space and block the dorsal and ventral rami of the spinal nerves after erector spinae block in different planes, and few studies have shown that it can block the sympathetic nerve [11]. However, OSTAPB only produces sensory blocks in the somatic branches of the spinal nerves. Thus, ESPB may have a potential analgesic mechanism for visceral pain and is expected to provide better analgesia than OSTAPB. The lack of difference in opioid consumption may have resulted from the three limited included studies. The incidence of PONV was also not significantly different between them. As only one study comparing ESPB and QLB in LC was included, further studies are required to answer the question of analgesic effect comparing ESPB and QLB.

This meta-analysis has some limitations that should be considered when interpreting the results. First, ESPB was conducted after anesthesia induction in some studies; therefore, any possible block failure could not be identified. Second, different block levels from T7 to T9, the concentration of different local anesthetics, and different types of analgesics may have influenced the results, and further studies are required to determine the optimal concentration, volume, and type of local anesthetic. Third, the heterogeneity cannot be ignored.

## Conclusions

ESPB plays an important role in the management of acute postoperative pain. To achieve opioid-sparing anesthesia, regional blocks such as ESPB should be advocated as part of multimodal analgesia for enhanced recovery after surgery. Further studies comparing ESPB, OSTAPB, and QLB are required to confirm their analgesic effects in LC.

## Supplementary Information

**Additional file 1.** Search strategy.

## Data Availability

The datasets generated and analyzed during the current study are available from the corresponding author on reasonable request.

## Abbreviations

LC: Laparoscopic cholecystectomy
ESPB: Erector spinae plane bolck
PRISMA: Preferred Reporting Items for Systematic Reviews and Meta Analyses
RCTs: randomized controlled trials
PONV: postoperative nausea and vomiting
RR: risk ratio
MD: mean difference
OSTAPB: oblique subcostal transversus abdominis plane block
QLB: quadratus Lumborum Block
