# Supplementary material for: Analgesic effect of erector spinae plane block in adults undergoing laparoscopic cholecystectomy: a systematic review and meta-analysis of randomized controlled trials
Source: BMC Anesthesiol. 2023 Jan 6;23:7. doi: 10.1186/s12871-023-01969-6 (PMC9817294; doi:10.1186/s12871-023-01969-6)
Supplement: Supplementary file 1 — Additional file 1. Search strategy. [file 12871_2023_1969_MOESM1_ESM.docx]

Additonal File 1. Search strategy

**Pubmed 36**	#1 Cholecystectomy, Laparoscopic [Mesh]<br>#2 Laparoscopic[Title/Abstract] OR Celioscopic [Title/Abstract]<br>#3 Cholecystectom*[Title/Abstract]<br>#4 #2 AND #3<br>#5 #1 OR #4<br>#6 erector spinae plane block[Title/Abstract] OR erector spinae plane[Title/Abstract] OR ESPB[Title/Abstract] OR ESP[Title/Abstract] OR ESB[Title/Abstract]<br>#7 #5 AND #6
**Cochrane 59**	#1 MESH descriptor "Cholecystectomy, Laparoscopic" explode all trees<br>#2 Laparoscopic OR Celioscopic<br>#3 Cholecystectom*<br>#4 #2 AND #3<br>#5 #1 OR #4<br>#6 erector spinae plane block OR erector spinae plane OR ESPB OR ESP OR ESB<br>#7 #5 AND #6
**Embase 53**	#1 'Cholecystectomy, Laparoscopic'/exp<br>#2 (Laparoscopic OR Celioscopic) AND Cholecystectom* : ti,ab<br>#3 #1 OR #2<br>#4 'erector spinae plane block'/exp<br>#5 erector spinae plane block OR erector spinae plane OR ESPB OR ESP OR ESB:ti,ab<br>#6 #4 OR #5<br>#7 #3 AND #6
**web of science 47**	#1 TS=（Laparoscopic OR Celioscopic）<br>#2 TS= Cholecystectom*<br>#3 #1 AND #2<br>#4 TS=（erector spinae plane block OR erector spinae plane OR ESPB OR ESP OR ESB）<br>#5 #3 AND #4
